# The transition to Xpert MTB/RIF ultra: diagnostic accuracy for pulmonary tuberculosis in Kampala, Uganda

**DOI:** 10.1186/s12879-020-05727-8

**Published:** 2021-01-11

**Authors:** A. Andama, D. Jaganath, R. Crowder, L. Asege, M. Nakaye, D. Katumba, J. Mukwatamundu, S. Mwebe, C. F. Semitala, W. Worodria, M. Joloba, S. Mohanty, A. Somoskovi, A. Cattamanchi

**Affiliations:** 1 Department of Internal Medicine, Makerere University College of Health Sciences, Ground Floor Pathology Building, Room A4, Kampala, Uganda; 2 Infectious Diseases Research Collaboration, Kampala, Uganda; 3 Department of Medicine, Division of Pulmonary & Critical Care Medicine, University of California San Francisco, San Francisco, California USA; 4 Center for Tuberculosis, University of California San Francisco, San Francisco, California USA; 5 Department of Pediatrics, Division of Pediatric Infectious Diseases, University of California San Francisco, San Francisco, California USA; 6 Mulago National Referral Hospital, Kampala, Uganda; 7 Department of Medical Microbiology, Makerere University College of Health Sciences, Kampala, Uganda; 8 Department of Chemical Engineering, Department of Materials Science Engineering, University of Utah, Salt Lake City, USA; 9 Global Good Intellectual Ventures Laboratory, Seattle, USA; 10 Center for Vulnerable Populations, Department of Medicine, University of California San Francisco, San Francisco, USA; 11 Curry International Tuberculosis Center, University of California San Francisco, San Francisco, USA

**Keywords:** Tuberculosis, Diagnostics, Xpert ultra, Xpert MTB/RIF, Uganda

## Abstract

**Background:**

The World Health Organization (WHO) has endorsed the next-generation Xpert MTB/RIF Ultra (Ultra) cartridge, and Uganda is currently transitioning from the older generation Xpert MTB/RIF (Xpert) cartridge to Ultra as the initial diagnostic test for pulmonary tuberculosis (TB). We assessed the diagnostic accuracy of Ultra for pulmonary TB among adults in Kampala, Uganda.

**Methods:**

We sampled adults referred for Xpert testing at two hospitals and a health center over a 12-month period. We enrolled adults with positive Xpert and a random 1:1 sample with negative Xpert results. Expectorated sputum was collected for Ultra, and for solid and liquid culture testing for Xpert-negative patients. We measured sensitivity and specificity of Ultra overall and by HIV status, prior history of TB, and hospitalization, in reference to Xpert and culture results. We also assessed how classification of results in the new “trace” category affects Ultra accuracy.

**Results:**

Among 698 participants included, 211 (30%) were HIV-positive and 336 (48%) had TB. The sensitivity of Ultra was 90.5% (95% CI 86.8–93.4) and specificity was 98.1% (95% CI 96.1–99.2). There were no significant differences in sensitivity and specificity by HIV status, prior history of TB or hospitalization. Xpert and Ultra results were concordant in 670 (96%) participants, with Ultra having a small reduction in specificity (difference 1.9, 95% CI 0.2 to 3.6, *p*=0.01). When “trace” results were considered positive for all patients, sensitivity increased by 2.1% (95% CI 0.3 to 3.9, *p*=0.01) without a significant reduction in specificity (− 0.8, 95% CI − 0.3 to 2.0, *p*=0.08).

**Conclusions:**

After 1 year of implementation, Ultra had similar performance to Xpert. Considering “trace” results to be positive in all patients increased case detection without significant loss of specificity. Longitudinal studies are needed to compare the benefit of greater diagnoses to the cost of overtreatment.

## Background

Nucleic acid amplification testing (NAAT) is recommended by the World Health Organization (WHO) as an initial test for pulmonary TB. In particular, WHO has endorsed Xpert MTB/RIF (Cepheid, Sunnyvale, CA USA), and 34.4 million Xpert MTB/RIF (Xpert) cartridges have been procured globally [1]. However, Xpert sensitivity has been sub-optimal for smear-negative pulmonary TB [2]. Consequently, the next-generation cartridge, Xpert MTB/RIF Ultra (Ultra), includes two multi-copy amplification targets, a larger DNA reaction chamber, and fully nested nucleic acid amplification to lower the limit of detection from 113 colony forming units (cfu)/mL to 16 cfu/ml, which is similar to growth detection [2]. Based on a multi-center assessment demonstrating increased sensitivity [3], the WHO recommended that Ultra replace Xpert as the initial diagnostic test [4].

Ultra cartridges are now expected to be widely used, including with the Gene Xpert Edge and Omni systems in lower-level facilities where patients more often seek initial medical care [5]. While the hope is that the lower limit of detection will increase case detection of patients with less advanced disease, there is also concern for reduced specificity and increased false-positive results, particularly among the category of “trace” results or in patients with past tuberculosis history [6].

During Uganda’s transition from Xpert to Ultra, we conducted a 12-month cross-sectional study to 1) assess the diagnostic accuracy of Ultra for pulmonary TB in adults, 2) determine changes in accuracy by HIV, prior history of TB, and inpatient status, and 3) characterize the impact of trace results on Ultra accuracy.

## Methods

### Study design and population

From April 2018 to April 2019, we conducted a cross sectional study of patients with presumed pulmonary TB at Kiruddu Hospital pulmonary ward and outpatient chest clinic, Mulago Hospital TB ward, and Kisenyi Health Center IV outpatient clinic in Kampala, Uganda. As previously described [7], we included adults (> 18 years) who were referred by clinicians for sputum-based TB testing (i.e.*,* Xpert). We excluded participants who received anti-TB treatment or antibiotics with anti-TB activity such as fluoroquinolones in the prior 12 months, or who refused or were unable to provide informed consent. To select participants, we reviewed the facility Xpert testing log daily. On each day, we enrolled all participants with Xpert-positive results and a random 1:1 sample of Xpert-negative patients identified from the log. The study and full protocol (available on request) was reviewed and approved by the Makerere University School of Medicine Research and Ethics Committee, the Uganda National Council for Science and Technology and the University of California San Francisco Committee on Human Research. This study was performed according to the Standards for Reporting of Diagnostic Accuracy Studies (STARD) guidelines [8].

### Study procedures

After consent, all eligible participants completed a survey on demographics and medical history. Each participant provided up to two sputum samples: one for Ultra testing (all participants) and one for solid (Lowenstein-Jensen [LJ]) and liquid (BACTEC MGIT 960, Becton Dickinson, USA) mycobacterial cultures (Xpert-negative participants only). Positive cultures were confirmed using Ziehl-Neelsen (ZN) staining and *M. tuberculosis* (MTB) complex speciation testing (MPT64TB Ag kit, SD Bioline, South Korea). Trained laboratory technologists conducted all TB testing following standard protocols for Xpert, Ultra [2], and mycobacterial culture [9]. Laboratory staff were blinded to Xpert results at the time of Ultra or culture testing. Participants with negative or unknown HIV status underwent HIV testing per national guidelines, and CD4 cell count was measured for HIV-positive participants. To assess any additional microbiological evidence for false-positive results, Determine TB LAM (Alere, Waltham, USA) was performed on urine samples following standard protocols [10].

#### Definitions

We used a composite microbiological reference standard. Participants were defined as having pulmonary TB if MTB was detected by sputum Xpert or mycobacterial culture (solid or liquid). We considered patients not to have pulmonary TB if Xpert was negative and at least two culture results (solid or liquid) were negative without contamination. For the primary analysis, an Ultra-trace result was defined as TB if the participant was HIV positive per WHO recommendations [11].

### Statistical approach

Descriptive statistics were used to summarize the demographic and clinical characteristics of study participants. The sensitivity and specificity of sputum Xpert Ultra was calculated with exact binomial 95% confidence interval (CI) using our reference standard definition of TB. We compared sensitivity and specificity by HIV status, CD4 cell count category, prior history of TB, and inpatient status using Chi-squared or Fisher’s Exact test as appropriate. We conducted a sensitivity analysis on Ultra trace results, comparing diagnostic accuracy if trace results were considered positive for all participants or no participants using McNemar’s paired test of proportions. We used STATA 15 (Stata Corp, College Station, TX, USA) to perform all analyses.

## Results

### Study population characteristics

**Fig. 1:**
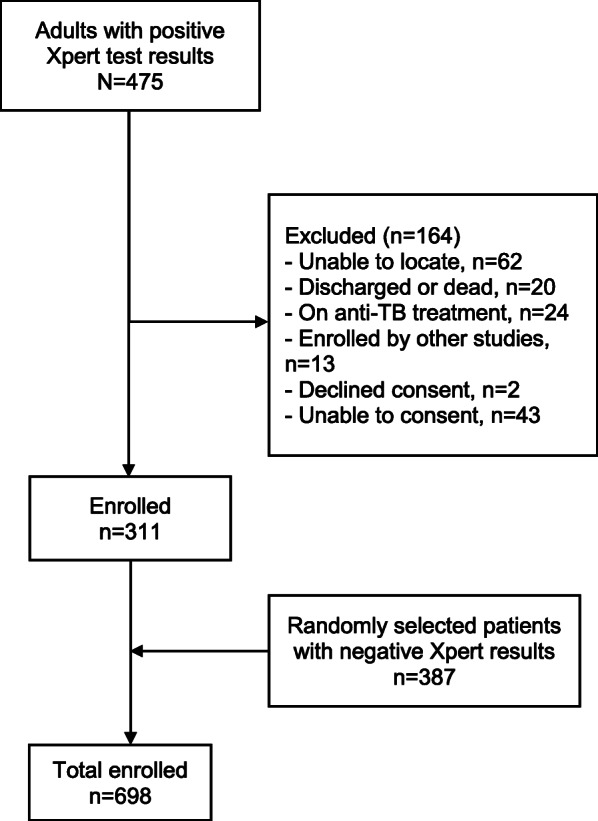
Flowchart of Participants

During the 12-month study period, we identified 475 patients with positive Xpert results in the facility testing logs. Of the 475 patients, 311 (65%) met eligibility criteria and were enrolled (Fig. 1). We randomly selected and enrolled 387 Xpert-negative participants, of whom 25 were subsequently found to be culture-positive for MTB. Thus, 698 participants (336 with and 362 without TB) were included in the final analysis. The median age was 32 years (IQR 25–40), 436 (62%) were male, over a third (42%) had BMI < 18.5, and 211 (30%) were HIV-positive, of whom 46 (22%) had CD4 cell count < 100 cells/μL (Table 1). Most participants were identified in the outpatient setting (*n*=620, 89%), and the median Karnofsky score was 80 (IQR 70–90).

**Table 1:** Patient characteristics (N=698)

Characteristic	N (%)
Age in years, median (IQR)	32 (25–40)
Male Sex	436 (62)
Cough ≥ 30 days	450 (65)
Fever ≥ 39 °C	20 (3)
Hospitalized	78 (11)
Smoking in last 30 days	95 (14)
Prior TB over 1 year ago	88 (13)
Karnofsky Score median (IQR) (*n*=323)	80 (70–90)
BMI < 18.5	295 (42)
HIV-positive	211 (30)
CD4 cell count < 100 cells/μL (*n*=211)	46 (22)

*IQR* Interquartile Range

### Diagnostic accuracy of Xpert ultra

Sputum Ultra had a sensitivity of 90.5% (95% CI 86.8 to 93.4) and specificity of 98.1% (95% CI 96.1–99.2) (Table 2**)**. Xpert and Ultra were concordant in 670/698 (96%) participants. Of the 28 discordant results, 14 (50%) were Ultra-positive and Xpert-negative (7 culture-positive), while 14 were Ultra-negative but Xpert-positive. Consequently, the sensitivity of Ultra was not significantly different from that of Xpert (90.5% vs. 92.6%, difference − 2.1, 95% CI − 5 to + 0.1, *p*=0.1). However, the seven false-positive Ultra results led to a small reduction in specificity of 1.9% (95% CI 0.2 to 3.6, *p*=0.01) compared to Xpert. Of note, 18 participants with positive cultures (5% of 336 TB cases) had negative Xpert and Ultra results.

**Table 2:** Sensitivity and Specificity of Sputum Xpert Ultra by HIV status and prior history of TB, in Kampala, Uganda

	Overall (***n***=698)^**a**^		Without prior TB (***n***=610)		With prior TB^**b**^ (***n***=88)	
	Sensitivity	Specificity	Sensitivity	Specificity	Sensitivity	Specificity
**Total Cohort** (*N*=336 with TB, 362 without TB)	90.5 (86.8, 93.4)	98.1 (96.1, 99.2)	90.9 (87.1, 93.9)	98.7 (96.6, 99.6)	85.7 (67.3, 96)	95 (86.1, 99)
**HIV-negative** (*N*=222 with TB, 212 without TB)	90.8 (86.3, 94.1)	98.8 (96.5, 99.8)	91.4 (87.0, 94.8)	99.5 (97.4, 100)	81.3 (54.4, 96)	94.6 (81.8, 99.3)
**HIV-positive** (*N*=86 with TB, 90 without TB)	89.8 (82.0, 95.0)	96.5 (91.2, 99.0)	89.5 (81.1, 95.1)	96.7 (90.6, 99.3)	91.7 (61.5, 99.8)	95.7 (78.1, 99.9)
**CD4 < 100** (*N*=25 with TB, 15 without TB)	96.2 (80.4, 99.9)	95.0 (75.1, 99.9)	96.0 (79.6, 99.9)	93.3 (68.1, 99.8)	100 (2.5, 100)	100 (47.8, 100)
**CD4 ≥100** (*N*=61 with TB, 75 without TB)	87.5 (77.6, 94.1)	96.8 (90.9, 99.3)	86.9 (75.8, 94.2)	97.3 (90.7, 99.7)	90.9 (58.7, 99.8)	94.4 (72.7, 99.9)

^a^Sensitivity and specificity presented as percentage with 95% CI

^b^Greater than one year from enrollment date

Among patients with positive Xpert and Ultra results, rifampicin resistance results were concordant in 285/297 (96%) patients. Three patients had rifampicin resistance detected by Ultra but not Xpert, and one patient had rifampicin resistance detected by Xpert but not Ultra. Rifampicin resistance results were indeterminate in two patients by Ultra (all due to trace results, Xpert RIF negative), and in one patient by Xpert (Ultra RIF negative). Two of the discordant results were missing on Ultra and were not resistant on Xpert, while the three results missing on Xpert were not resistant on Ultra.

### Subgroup analysis of ultra performance

As shown in Table 2, Ultra sensitivity and specificity did not differ by HIV status (sensitivity difference 0.96, 95% CI − 6.1 to 8.0, *p*=0.79; specificity difference − 2.3, 95% CI − 6.0 to 1.33, *p*=0.1). Ultra sensitivity was also similar among HIV-positive patients with CD4 cell count < 100 cells/μL vs. higher CD4 cell counts (difference 8.6, 95% CI − 2.0 to 19.3, *p*=0.2). There was also no difference among patients with and without a prior history of TB (sensitivity 85.7% vs. 90.9%, difference − 5.2, 95% CI − 18.5 to 8.2, *p*=0.37; specificity 95.0% vs. 98.7%, difference − 3.7, 95% CI − 9.3 to 2.0, *p*=0.06) or among inpatients vs. outpatients (sensitivity 87.2% vs. 90.9%, difference 3.7, 95% CI − 7.3 to 14.7, *p*=0.46; specificity 100% vs 97.8%, difference 2.2, 95% CI 0.6 to 3.7, *p*=0.35).

### False-positive and trace ultra results

Of the seven patients with false-positive Ultra results, four were HIV-positive (including one with CD4 cell count < 100 cells/μL), of which one had a prior history of TB and all had trace results. Of the remaining three HIV negative participants, two had a prior history of TB.

There were 21 trace Ultra results. Of the 11 HIV-positive patients with trace results (Table 3), eight had microbiological evidence of TB (two Xpert-positive with a very low semi-quantitative result, five culture-positive and one urine Determine TB LAM-positive). Of the 10 HIV-negative patients with trace results, 8 had microbiological evidence of TB (four Xpert positive with low [*N*=1] or very low [*N*=3] semi-quantitative results, three culture-positive, and one urine Determine TB LAM-positive). Thus, 16 of 21 (76%) patients with trace results had microbiologic evidence of TB.

**Table 3:** HIV positive participants with Ultra Trace results (*N*=11)

TB status	Xpert MTB/RIF result	CD4 count < 100?	Determine TB LAM result	Low Body Mass Index^**a**^ (BMI)	Prior history of TB
Negative	Negative	No	Negative	No	No
Negative	Negative	Yes	Positive	No	No
Negative	Negative	No	Negative	No	No
Negative	Negative	No	Negative	Yes	Yes
Confirmed TB	Negative^b^	No	Negative	Yes	Yes
Confirmed TB	Negative^b^	No	Negative	No	Yes
Confirmed TB	Negative^b^	No	Negative	No	No
Confirmed TB	Negative^b^	No	Negative	No	No
Confirmed TB	Negative^b^	Yes	Positive	Yes	No
Confirmed TB	Very low	No	Negative	No	Yes
Confirmed TB	Very low	No	Negative	No	No

^a^BMI < 18.5 kg/m^2^

^b^Solid or liquid culture positive

We examined how classification of trace results impacted Ultra accuracy (Table 4). When trace results were defined as positive for all patients, Ultra sensitivity and specificity were 92.6% (95% CI 89.2 to 95.1) and 97.2% (95% CI 95.0 to 98.7), respectively, representing a 2.1% increase in sensitivity (95% CI 0.3 to 3.9, *p*=0.01) and a non-significant 0.8% reduction in specificity (95% CI − 0.3 to 2.0, *p*=0.08) compared to when trace results were considered to be positive only in HIV-positive patients as per WHO guidelines. When trace results were defined to be negative for all patients, Ultra sensitivity and specificity were 88.4% (95% CI 84.5 to 91.6) and 99.2% (95%CI 97.6 to 99.8), respectively, representing a 2.1% reduction in sensitivity (95% CI − 3.9 to − 0.3, *p*=0.01) and non-significant 1.1% increase in specificity (95% CI − 0.2 to 2.5, *p*=0.05) compared to the current guidelines.

**Table 4:** Sensitivity analysis of Ultra performance by Trace status

	Sensitivity (%, 95 CI)	% Difference (95% CI)^**b**^	Specificity (%, 95 CI)	% Difference (95% CI)^**b**^
**Trace Positive in HIV infection**^**a**^	90.5% (86.8–93.4)	–	98.1% (96.1–99.2)	–
**Trace Positive**	92.6% (89.2–95.1)	2.1% (0.3 to 3.9)	97.2% (95.0–98.7)	−0.8 (−0.3 to 2.0)
**Trace Negative**	88.4% (84.5–91.6)	−2.1% (−3.9 to −0.3)	99.2% (97.6–99.8)	1.1 (− 0.2 to 2.5)

^a^Current WHO recommendations [4]

^b^Compared to current recommendation of trace conditional positive

## Discussion

As Uganda is transitioning from Xpert to Ultra, we found that Xpert Ultra had high sensitivity and specificity for pulmonary TB overall (90.5% sensitive and 98.1% specific) and across key sub-groups defined by HIV status, prior history of TB, and hospitalization. Expanding the definition of a positive test to include trace results among HIV-negative patients increased Ultra sensitivity without a significant loss of specificity. Our findings suggest that as Ultra replaces Xpert in Kampala for routine TB testing at health facilities, diagnostic accuracy may remain stable overall, but that expanding the definition of a positive test to include all trace results could increase case detection.

The accuracy of Ultra for pulmonary TB diagnosis was similar to previous studies. A recent meta-analysis of 10 studies found that Ultra could detect pulmonary TB with a sensitivity of 88.5% and specificity of 96.7% [12]. Among HIV-positive patients, we also found a similar Ultra sensitivity to other studies in HIV-endemic settings that found an 87–90% sensitivity without significant difference compared to HIV-negative patients [3, 13]. Some studies have found that a previous history of TB reduced Ultra specificity [3, 14, 15], whereas we did not perhaps because we excluded patients who had TB in the past 1 year. The role of hospitalization has not been previously evaluated and we did not find a significant difference in inpatients vs. outpatients.

We did not find a significant difference in sensitivity of Xpert vs. Ultra. This is in contrast to a multicenter study that found Ultra was 5.4% more sensitive overall and 13% more sensitive in HIV-positive patients [3]. The lower yield in our study is likely due to the small proportion of culture-positive patients that were not detected by Xpert (for example, 7% in our study compared to 17% in Dorman et al. [3]), and potentially the inclusion of Xpert in our reference standard. At the same time, multiple studies in Brazil and South Africa also had high concordance between Ultra and Xpert without a significant difference in overall sensitivity [13, 15, 16]. Nonetheless, this finding is surprising given that we had a high prevalence of HIV-positive patients and outpatients, both of whom would be expected to have lower bacillary burden and greater yield with Ultra over Xpert. In addition, 5% of culture-confirmed cases were both Ultra- and Xpert-negative. This underscores the continued need for novel diagnostics that can increase the yield of TB case detection in patients with paucibacillary disease.

The reduction in specificity of Ultra vs. Xpert has been documented in several studies [3, 13, 15, 17]. Four of seven false positive results were trace results among HIV positive patients, of which one had additional evidence of TB disease with a positive Determine TB LAM result. While this is consistent with past studies that found the majority of false positives were due to trace results [3, 13], we had few overall false positives and only three had a prior history of TB. As a consequence, we did not find a significant increase in specificity when trace results were defined as negative. Conversely, expanding the definition of a positive test to include trace results in HIV-negative patients increased sensitivity without a significant loss of specificity, because the majority of HIV-negative patients with trace results had microbiologically-confirmed TB.

The decision to define trace results as positive or negative requires a balance between greater case detection and cost of overtreatment [6]. A modeling study found that while Ultra trace results increased overtreatment by more than 50%, it also averted deaths by that amount [18]. The authors further concluded that Ultra and trace results will have the greatest benefit in settings with high HIV and TB prevalence and TB mortality. As Uganda is one of those settings, further cost-effectiveness studies are needed to guide the role of trace results, especially in facilities where health workers may not have access to culture or other testing to confirm the diagnosis.

To evaluate the performance of Ultra in Uganda, we enrolled a sample from three major medical centers in Kampala with high HIV prevalence [19]. However, this study has some limitations. We did not confirm positive sputum Xpert results with culture, and did not confirm rifampin resistance with drug susceptibility testing. However, Xpert has been shown to have very high specificity (> 98%) for TB and rifampin resistance [20]. As smear microscopy was not done on Xpert positive samples, we could not stratify Xpert Ultra performance by smear status. We also did not have the ability to have follow-up visits to repeat Ultra testing on trace results to assess additional yield. In addition, follow-up visits would have been helpful to further characterize patients with both false-positive and trace Ultra results.

## Conclusions

As Uganda and similar high burden settings transition to Ultra, it is important to characterize the diagnostic accuracy and anticipate any change compared to Xpert for appropriate resource allocation to manage TB cases. Based on two hospitals and a health center in Kampala, Ultra has high accuracy for pulmonary TB among adults with and without HIV. Longitudinal studies are needed to characterize the ongoing impact of Xpert Ultra and trace results on TB case detection and management.

## Data Availability

The datasets used and/or analyzed during the current study are available from the corresponding author on reasonable request.

## Abbreviations

CI: Confidence Interval
HIV: Human Immunodeficiency Virus
IQR: Interquartile Range
MTB: *Mycobacterium tuberculosis*
NAAT: Nucleic acid amplification testing
TB: Tuberculosis
WHO: World Health Organization
Ultra: Xpert MTB/RIF Ultra
Xpert: Xpert MTB/RIF
